# Long Non-coding RNAs as Local Regulators of Pancreatic Islet Transcription Factor Genes

**DOI:** 10.3389/fgene.2018.00524

**Published:** 2018-11-06

**Authors:** Berta Font-Cunill, Luis Arnes, Jorge Ferrer, Lori Sussel, Anthony Beucher

**Affiliations:** ^1^Department of Medicine, Imperial College London, London, United Kingdom; ^2^Department of Systems Biology, Columbia University Medical Center, New York, NY, United States; ^3^Department of Biomedical Informatics, Columbia University Medical Center, New York, NY, United States; ^4^CIBER de Diabetes y Enfermedades Metabólicas Asociadas, Barcelona, Spain; ^5^Department of Genetics and Development, Columbia University Medical Center, New York, NY, United States; ^6^Barbara Davis Center, University of Colorado Anschutz Medical Campus, Aurora, CO, United States

**Keywords:** long non-coding RNAs, transcription factors, pancreatic islets, β cells, *cis*-regulation

## Abstract

The transcriptional programs of differentiated cells are tightly regulated by interactions between cell type-specific transcription factors and *cis*-regulatory elements. Long non-coding RNAs (lncRNAs) have emerged as additional regulators of gene transcription. Current evidence indicates that lncRNAs are a very heterogeneous group of molecules. For example, selected lncRNAs have been shown to regulate gene expression in *cis* or *trans*, although in most cases the precise underlying molecular mechanisms is unknown. Recent studies have uncovered a large number of lncRNAs that are selectively expressed in pancreatic islet cells, some of which were shown to regulate β cell transcriptional programs. A subset of such islet lncRNAs appears to control the expression of β cell-specific transcription factor (TF) genes by local *cis*-regulation. In this review, we discuss current knowledge of molecular mechanisms underlying *cis*-regulatory lncRNAs and discuss challenges involved in using genetic perturbations to define their function. We then discuss known examples of pancreatic islet lncRNAs that appear to exert *cis*-regulation of TF genes. We propose that *cis*-regulatory lncRNAs could represent a molecular target for modulation of diabetes-relevant genes.

## Introduction

Cell-specific genome regulation in pancreatic islet cells is driven by combinations of transcription factors (TFs) that interact with *cis*-regulatory elements. Understanding islet-specific transcriptional programs is critically important for strategies to derive β cells for the treatment of type 1 diabetes, as well as for efforts to understand the pathophysiology of monogenic and type 2 diabetes.

In recent years, several lines of evidence have pointed to a potentially important role of long non-coding RNAs (lncRNAs) in the regulation of gene transcription (Rinn and Chang, 2012). LncRNAs are transcripts >200 nucleotides in length that do not encode for proteins. As a result of this broad definition, lncRNAs represent an extremely heterogeneous group of transcripts, a subset of which modulate gene expression through varied mechanisms, including regulation of epigenetic modifications, transcriptional initiation, splicing, mRNA stability and translation (Rinn et al., 2007; Zhao et al., 2008; Tripathi et al., 2010; Carrieri et al., 2012; Kretz et al., 2013; Xing et al., 2014; Hosono et al., 2017; Kopp and Mendell, 2018). This realization poses a need to understand how specific lncRNA subtypes influence regulatory programs.

Several studies have described thousands of lncRNAs expressed in human and mouse pancreatic islets (Ku et al., 2012; Morán et al., 2012; Bramswig et al., 2013; Akerman et al., 2017; Motterle et al., 2017), and have been recently thoroughly reviewed (Pullen and Rutter, 2014; Motterle et al., 2016; Mirza et al., 2017; Singer and Sussel, 2018). The current review focuses on a discrete subset of lncRNAs that have been shown to modulate the transcription of nearby genes, many of which encode for transcription factors. Understanding the *cis*-regulatory function of certain lncRNAs is likely to provide new insights into genome regulation, and could reveal targets for gene-specific manipulation. However, the analysis of *cis*-regulatory lncRNAs poses significant experimental challenges. We provide an overview of recent progress in the analysis of *cis*-regulatory lncRNAs, and discuss obstacles to understand their function. We also discuss specific examples in the recent literature of *cis*-regulatory pancreatic islet lncRNAs.

## *Cis*-Regulatory lncRNAs

**FIGURE 1:**
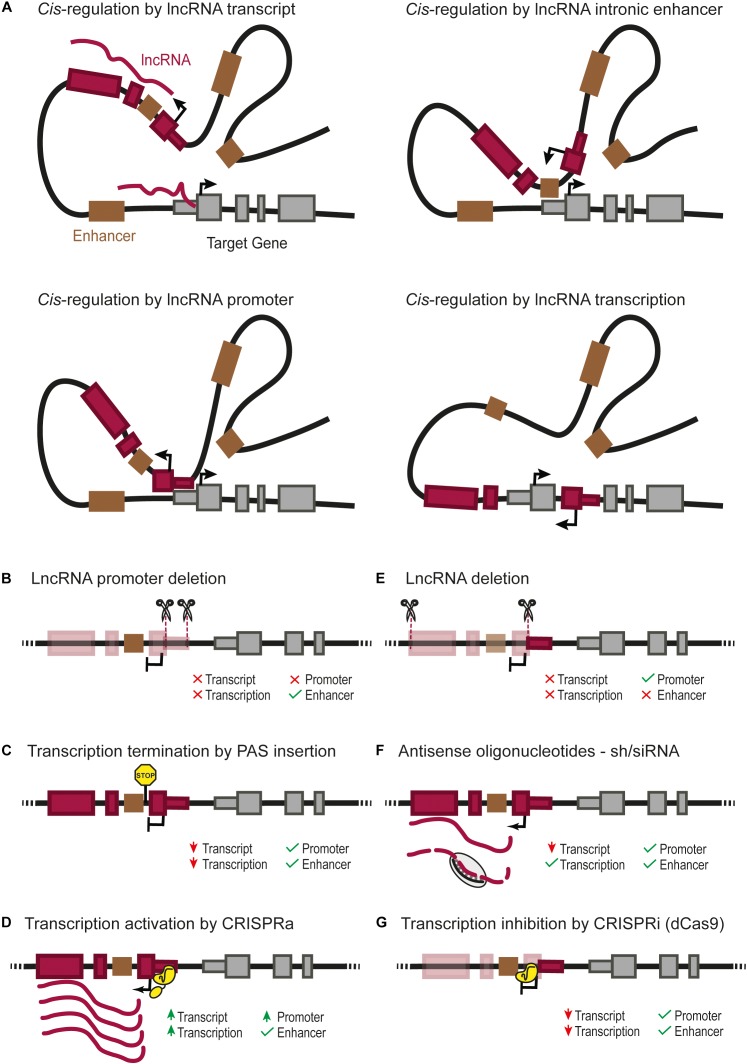
**(A)** Four possible *cis*-regulatory mechanisms mediated by lncRNA genes. *Cis*-regulation of nearby genes can be achieved directly by the lncRNA transcript, by *cis*-regulatory elements overlapping with the lncRNA gene such as an enhancer (Groff et al., 2016), or the lncRNA promoter itself (Engreitz et al., 2016; Paralkar et al., 2016), or by the transcription of the lncRNA (Latos et al., 2012). Different types of genetic perturbation can provide insights into the underlying mechanisms, such as: **(B)** deletion of the lncRNA promoter, which can affect the transcription of the regulated gene due to the lack of lncRNA transcript, transcription or absence of direct *cis*-regulation by the lncRNA promoter (Engreitz et al., 2016; Paralkar et al., 2016); **(C)** insertion of a poly-adenylation signal (PAS) downstream of the transcription start site, which prevents the transcription of the lncRNA through downstream DNA sequences (Sleutels et al., 2002; Ohhata et al., 2007; Yin et al., 2015; Anderson et al., 2016; Engreitz et al., 2016; Paralkar et al., 2016); **(D)** over-expression of a lncRNA from its endogenous locus can be achieved by CRISPR-activation (CRISPRa) (Gilbert et al., 2014; Konermann et al., 2015; Joung et al., 2017); it should be noted that the CRISPR-activation of the lncRNA promoter can also increase a direct *cis*-regulatory activity of the lncRNA promoter; **(E)** deletion of the full lncRNA transcript, which leaves the lncRNA promoter as a potential *cis*-regulatory element; note that deletion of several exons of the lncRNA will also remove any intronic enhancer (Groff et al., 2016); **(F)** lncRNA knock-down using short hairpin RNA (siRNA/shRNA) or antisense oligonucleotides, testing the functionality of the lncRNA transcript itself; **(G)** RNA Polymerase II “roadblock” by CRISPR-dCas9 downstream of the transcriptional start site of the lncRNA (Gilbert et al., 2013; Qi et al., 2013); this selectively blocks the transcription of the lncRNA, helping to discriminate a *cis*-regulatory function from the transcription/transcript or from genomic *cis*-regulatory elements.

Emerging evidence points to the existence of lncRNAs that regulate nearby genes in *cis*. In contrast to mRNAs that need to be translated in the cytoplasm to produce functional proteins, lncRNAs can exist in their functional conformation immediately after their transcription, and can thus theoretically exert their function in any cellular compartment, including their site of transcription. Several studies have thus revealed lncRNAs that exert *cis-*regulation of nearby regulatory elements such as promoters and enhancers (Figure 1A; Wang et al., 2011; Latos et al., 2012; Postepska-Igielska et al., 2015; Yin et al., 2015; Anderson et al., 2016; Kotzin et al., 2016; Luo et al., 2016).

The precise number of *cis*-regulatory lncRNAs is unknown. Cell fractionation experiments have revealed that a large portion of lncRNAs is enriched in the nucleus and tightly bound to chromatin (Engreitz et al., 2016; Werner et al., 2017). The expression of chromatin-enriched RNAs has been shown to exhibit higher correlation with their neighboring genes than other lncRNAs, suggesting that many of them could be *cis*-regulators (Werner et al., 2017). A distinct subset of nuclear lncRNAs are transcribed from active enhancers, and are known as enhancer RNAs (eRNAs). These are often unstable and non-polyadenylated transcripts, and their expression often correlates with the mRNA of the enhancer’s target gene (Kim et al., 2010). In some cases, eRNA transcripts have been shown to regulate the activity of target genes (Bose et al., 2017). However, whether most eRNA transcripts exert true *cis*-regulatory effects is currently a matter of debate. Furthermore, the distinction between enhancers and promoters is often not unequivocal (Carelli et al., 2018). Thus, although it is useful to classify a subset of lncRNAs as eRNAs, it is possible that lncRNAs that originate from either promoters or enhancers can regulate the expression of nearby genes in *cis*. The current challenge, therefore, is to define which pairs of lncRNAs and adjacent coding genes show correlated transcription due to true *cis*-regulatory relationships.

## Genetic Approaches to Identify *cis*-Regulatory lncRNAs

Genetic tools have been used to discriminate *cis* vs. *trans* regulation by lncRNAs. Some studies, for example, have used compound heterozygote mice where one chromosome contains an inactive lncRNA allele while the other has a null allele of the coding gene that is regulated by the lncRNA (Dimitrova et al., 2014; Anderson et al., 2016; Kotzin et al., 2016; Herriges et al., 2017). For example, Anderson et al. (2016) showed that homozygous insertion of a premature transcription termination signal to inactivate *Upperhand* (*Uph*), a lncRNA expressed upstream of the cardiac-specific TF *Hand2*, results in ventricular hypoplasia and embryonic lethality, a phenotype similar to the *Hand2* gene knockout. While heterozygous mice for the lncRNA or TF are both viable, compound heterozygous mice (*Uph*^+/-^;*Hand2*^+/-^) display the same phenotype as the *Uph*^-/-^ mice with a nearly complete absence of Hand2 expression. This result was interpreted as an indication that the inactivation of *Uph* lncRNA transcription caused decreased expression of *Hand2* in *cis*, thereby suggesting that *Uph* is a *cis*-regulator of the cardiac TF *Hand2*.

The *cis*-regulatory function of a lncRNA can also be assessed by creating a heterozygous mutation of the lncRNA and then use allelic markers to distinguish the expression of the nearby target gene in the mutated and wild type chromosomes. This can be implemented in hybrid mouse strains that have single nucleotide polymorphisms (SNPs) within the target gene (Engreitz et al., 2016). Heterozygous inactivation of the *cis*-regulatory lncRNA gene should thus result in allelic imbalance of the target gene, with altered expression of transcripts derived from the same chromosome as the mutations.

The abovementioned approaches can provide genetic evidence that is consistent with *cis*-regulatory effects of a lncRNA. However, they do not always provide conclusive evidence that the lncRNA transcript is exerting the effect. The distinction between different candidate mechanisms involved can be addressed with complementary genetic approaches that are summarized in Figures 1B–G.

## Experimental Challenges to Understand Different Classes of *cis*-Regulatory lncRNAs

LncRNAs can overlap *cis*-regulatory elements such as enhancers (Figure 1A). In such cases, the effect of deleting the lncRNA can result from the deletion of one or more enhancers, even if the lncRNA itself has no function, or if there is a *cis* effect of both the lncRNA and the enhancer (Huarte et al., 2010; Dimitrova et al., 2014; Groff et al., 2016). In certain contexts, a lncRNA and *cis*-regulatory elements have opposite roles. This is true for *Haunt*, a lncRNA expressed more than 40 kb upstream of the *HOXA* cluster (Yin et al., 2015). Deletion and premature transcription termination of *Haunt* result in opposite effects. Complementary genetic models used in this study showed that the Haunt transcript directly represses *HOXA* enhancers after exposure to retinoic acid. Thus, *cis*-regulatory lncRNAs can buffer the expression of their target genes by direct negative regulation of their underlying *cis*-regulatory elements. The complexity of such studies reveals the peril of using a single perturbation model to study the function of *cis*-regulatory lncRNAs.

In some cases, the promoter of a lncRNA gene can act as a functional *cis*-regulatory element for another gene (Figure 1A; Cho et al., 2018). The comparison of promoter deletions vs. premature transcription termination of the lncRNA by insertion of a polyadenylation signal in *Lockd* or *Bendr* lncRNA genes showed that the promoter of a lncRNA is capable of exerting *cis*-regulation of a nearby gene while the transcript itself is not visibly functional (Figures 1B,C; Engreitz et al., 2016; Paralkar et al., 2016). Nevertheless, even in these cases, it is difficult to entirely rule out the function of lncRNA transcripts, since it is still possible that a short exon transcribed before the polyadenylation signal would be the functional part of the lncRNA transcript.

Gain-of-function studies have also provided useful insights into lncRNA functions (Shechner et al., 2015; Joung et al., 2017; Bester et al., 2018). Ectopic expression of lncRNAs, however, also comes with caveats. *Cis*-regulatory lncRNAs sometimes need to act near their transcription sites, in which case they need to be activated from their endogenous genes, rather than from ectopic artificial transgenes (Dimitrova et al., 2014). CRISPR activation (CRISPRa) could be a powerful tool to accomplish this (Figure 1D; Gilbert et al., 2014; Konermann et al., 2015). However, it remains possible that any downstream effect of activating a lncRNA can be attributed either to the lncRNA transcript itself or to a direct *cis*-regulatory effect of activating the lncRNA promoter. To this end, it is important to include proper controls such as combining CRISPRa of the lncRNA promoter with the silencing of the lncRNA transcript (Figures 1D–G; Joung et al., 2017). Alternatively, CRISPR/dCas9 technology can be used to artificially tether a lncRNA to a specific locus and test whether the lncRNA function is recapitulated by the exogenous transcript (Shechner et al., 2015). This technique has the power to complement other methods to demonstrate a function intrinsic to the lncRNA transcript, independent from any genomic element.

Several lines of evidence suggest that the *cis*-regulatory effect of some lncRNAs could result from the act of transcription or transcriptional splicing rather than from the transcript molecule or the promoter activity (Figure 1A; Latos et al., 2012; Engreitz et al., 2016). These mechanisms need to be tested through approaches that specifically disrupt transcription or splicing (Figures 1C,G).

In summary, existing evidence indicates that the *cis*-regulatory function of lncRNA genes can be attributed to several processes, including an effect of the transcript, its transcription, splicing, or the activity of its promoter. All of these need to be carefully distinguished from overlapping DNA *cis*-regulatory functions that are unrelated to the lncRNA gene. This warrants a need to investigate *cis*-regulatory lncRNAs with complementary models that consider these possible genetic mechanisms.

## Molecular Mechanisms Underlying *cis*-Regulatory lncRNAs

**FIGURE 2:**
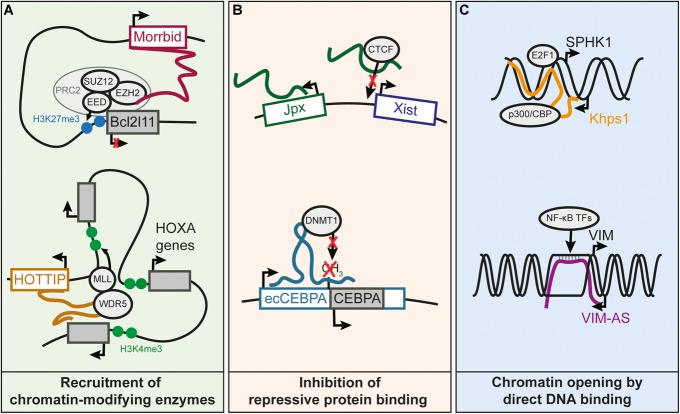
Known molecular mechanisms underlying *cis*-regulation by lncRNAs. **(A)** Recruitment of chromatin-modifying enzymes by *cis*-regulatory lncRNAs. Morrbid represses the *Bcl2l11* gene by recruiting the PRC2 complex to the *Bcl2l11* promoter (Kotzin et al., 2016). HOTTIP recruits the WDR5 subunit of the MLL complex, resulting in the deposition of the H3K4me3 mark and subsequent activation of the HOXA distal genes (Wang et al., 2011). **(B)** LncRNA prevents binding of an inhibitory protein to a promoter, which results in gene activation. Jpx lncRNA competes with DNA for the binding of CTCF on *Xist* promoter, and this enables activation of *Xist* expression (Sun et al., 2013). Transcription of ecCEBPA, a sense-overlapping lncRNA of the *CEBPA* gene, prevents the silencing of *CEBPA* by inhibiting DNMT1-dependent DNA methylation of *CEBPA* promoter (Di Ruscio et al., 2013). **(C)** Increased chromatin accessibility by DNA binding of lncRNA. The Khps1 lncRNA forms a triple helix with the promoter of *SPHK1*, which leads to the successive recruitment of p300/CBP and H3K27 acetylation of *SPHK1* promoter, facilitating the binding of TFs on the *SPHK1* promoter (Postepska-Igielska et al., 2015). VIM-AS lncRNA sustains the expression of the *VIM* gene by forming an R-loop at the *VIM* promoter (Boque-Sastre et al., 2015). The R-loop helps keeping the chromatin open, allowing TFs of the NF-kB pathway to bind to the *VIM* promoter and activate *VIM* expression.

*Cis*-regulation mediated by lncRNA genes can be achieved through various molecular mechanisms, illustrated by several well characterized cases. *XIST* is an example of a repressive *cis*-regulatory lncRNA in which the molecular mechanisms have been thoroughly characterized (da Rocha and Heard, 2017). XIST is responsible for random inactivation of the X chromosome. It is known to recruit Polycomb Repressive Complex 2 (PRC2) near the *Xist* locus on chromosome X, triggering chromosomal condensation (Zhao et al., 2008). *Cis*-regulatory lncRNAs have also been shown to interact with the members of PRC2 such as the lncRNA Morrbid (Figure 2A), located over 100 kb from *Bcl2l11* which binds and represses the promoter of *Bcl2l11* in *cis* (Klattenhoff et al., 2013; Kotzin et al., 2016). Other *cis*-regulatory lncRNAs appear to promote the formation of heterochromatin at imprinted loci, through binding of the histone methyltransferase G9A (Nagano et al., 2008).

Several lncRNAs have been shown to recruit transcriptional activating complexes to adjacent genes (Wang et al., 2011; Yang et al., 2014; Bose et al., 2017). The histone lysine-methyltransferase WDR5, a component the MLL complex responsible for H3K4me2/3 deposition, contains an RNA-binding domain that can bind lncRNAs (Yang et al., 2014). For instance, it was reported that activation of the 5^′^ genes of the *HOXA* cluster requires WDR5 recruitment by the HOTTIP lncRNA, also encoded in this locus (Wang et al., 2011; Figure 2A). Other lncRNAs have been shown to promote interactions between enhancers and their targets promoters, thereby sustaining transcriptional activity (Luo et al., 2016; Akerman et al., 2017). So far, little is known on the mechanism explaining how some lncRNAs can stabilize or enhance the interaction of a promoter with its enhancers. Recruitment of Mediator subunits is one of the suggested mechanisms (Luo et al., 2016), although further research is needed to understand how lncRNAs could modulate the Mediator complex or any other proteins involved in chromatin structure.

Transcriptional activity can also be maintained by preventing DNA methylation through interference with DNA methyltransferases (Di Ruscio et al., 2013), or by competing with DNA for binding of repressor proteins (Figure 2B; Sun et al., 2013).

Some lncRNAs can function through direct interaction with DNA to create R-loops (i.e., DNA-RNA hybrids through Watson-Crick base pairing) or DNA-RNA triplex structures (Figure 2C; Boque-Sastre et al., 2015; Postepska-Igielska et al., 2015; Li et al., 2016). R-loop formation could help maintain open chromatin at promoters (Boque-Sastre et al., 2015). In contrast to R-loops, the RNA-DNA triplex occurs when RNA interacts with the major groove of the DNA double helix (Li et al., 2016). RNA-DNA triplex interactions have been proposed for lncRNAs regulating their targets in *trans* or *cis.* For example, the KHPS1 antisense lncRNA forms a triple helix with the *SPHK1* promoter to facilitate the binding of E2F2 that in turn activates both *SPHK1* and its antisense lncRNA (Mondal et al., 2015; Postepska-Igielska et al., 2015). In summary, this non-exhaustive list of mechanisms illustrates a wide range of processes through which lncRNAs can be involved in gene *cis*-regulation.

## Co-Regulation of Islet β Cell Programs by TFs and lncRNAs

Human pancreatic islets transcribe well over 1000 lncRNAs, many of which are highly specific to islet cells (Morán et al., 2012). To explore the functionality of these lncRNAs, Akerman et al. (2017) performed a loss-of-function screen of 12 β cell-enriched lncRNAs in a human β cell line named EndoC-βH1 (Ravassard et al., 2011). Transcriptional profiling of cells after lncRNA inhibition revealed several lncRNAs with loss of function phenotypes consistent with a gene regulatory function. Comparison of transcriptional profiles after knock-down of β cell-specific lncRNAs and TFs (PDX1, HNF1A, MAFB, GLIS3, and *NKX2-2*) showed that genes regulated by β cell TFs are often also regulated by β cell lncRNAs. Moreover, the genes that were regulated by several β cell lncRNAs were in turn associated with clusters of enhancers that are highly bound by cell type-specific TFs (Akerman et al., 2017). Taken together, these results indicated that some cell-specific lncRNAs and TFs regulate common networks. To independently validate this finding, Akerman et al. (2017) performed weighted gene co-expression network analysis (WGCNA) using RNA-seq data from a panel of human islets. This approach uses gene expression correlation across samples to group genes into modules that form part of common transcriptional programs. This revealed specific expression modules that were co-enriched in cell-specific lncRNAs, islet TFs and genes regulated by enhancer clusters (Akerman et al., 2017). Interestingly, knockdown of lncRNAs from these modules led to downregulation of other genes in the same modules, which suggests that the lncRNAs did indeed exert a regulatory function in these cell-specific programs. Taken together, these independent lines of evidence revealed tissue-specific lncRNAs that modulate cell type-specific gene programs.

The mechanisms by which these islet lncRNAs regulate gene expression are still largely unknown. Potential scenarios include the possibility that lncRNAs act in *cis* or *trans*. Among the seven lncRNAs that regulate enhancer cluster-associated genes, HI-LNC30, HI-LNC12, HI-LNC78 (also known as TUNAR; (Lin et al., 2014) and HI-LNC80 (also known as OLMALINC; (Mills et al., 2015) had no apparent effect on adjacent genes, arguing against a *cis*-regulatory mechanism. However, three lncRNAs regulating β cell enriched genes were shown to also control the expression of their neighboring gene. Akerman et al. (2017) showed that knock-down of the *PLUTO* lncRNA caused decreased expression of the adjacent gene *PDX1*. Similarly, knockdown of HI-LNC15, the human homolog of *βlinc1* (Arnes et al., 2016), caused decreased expression of the adjacent gene *NKX2-2*. In both cases, knockdown of the lncRNA and the adjacent TF gene resulted in highly correlated changes in differential expression. Thus, it is highly probable that both *PLUTO* and *HI-LNC15* modulate in *cis* the expression of *PDX1* and *NKX2-2*, respectively, and elicit their regulatory function through this mechanism.

## *PLUTO* Regulates 3D Chromatin Structure at the *PDX1* Locus

PDX1 is a key transcriptional regulator of pancreas development and β cell function. It is required for the initial stages of pancreas formation (Jonsson et al., 1994; Stoffers et al., 1997), but is also necessary for the correct function of mature β cells (Ahlgren et al., 1998). *PDX1* expression is regulated by a cluster of upstream enhancers (Gerrish et al., 2000; Pasquali et al., 2014). LncRNA PLUTO, which stands for *PDX1 Locus Upstream Transcript*, is a multiexonic gene that originates approximately 3 kb upstream from *PDX1*, and is transcribed in the opposite strand through a ∼100 kb region that contains a cluster of active islet enhancers (Akerman et al., 2017). The positive regulatory effect of *PLUTO* on *PDX1* was shown in human β cell lines as well as in human islets, and it can be elicited through short hairpin RNA (shRNA) knock-down or transcription interference of *PLUTO* with CRISPR/dCas9. Moreover, the genes regulated by PLUTO are essentially the same as the genes regulated by PDX1, suggesting that PLUTO regulates β cell gene expression indirectly through the regulation of *PDX1* (Akerman et al., 2017).

Conformation capture experiments indicated that PLUTO promotes interactions of nearby clustered enhancers and the *PDX1* promoter. This indicates that PLUTO has a structural function in the regulation of the *PDX1* locus (Akerman et al., 2017). Very recently, Amaral et al. (2018) identified a sub-class of lncRNAs that are transcribed from evolutionarily conserved promoters, frequently found in the vicinity of developmental TFs and with an expression correlating with the nearby TF gene (Amaral et al., 2018). Many of these lncRNAs were found to be located near topologically associating domain (TAD) boundaries and overlap CTCF binding sites, pointing to a possible role in genomic 3D organization (Amaral et al., 2018). Thus, *PLUTO* may be an example of a broad class of lncRNAs that exhibits an analogous function of regulating 3D chromatin structure of genes important for lineage-specific functions.

Interestingly, both *PLUTO* and *PDX1* are down-regulated in islets from donors with type 2 diabetes or impaired glucose tolerance (Akerman et al., 2017). *PLUTO* lncRNA thus emerges as a *cis*-regulatory lncRNA that is a candidate player in β cell differentiation, with a potential involvement in diabetes.

## Genetic Analysis of *βlinc1* Points to a Cell-Specific Differentiation Function

*NKX2-2* is another essential transcriptional regulator of islet cell differentiation in the embryo, and β cell function in the adult (Papizan et al., 2011; Gutiérrez et al., 2017). Mice lacking *Nkx2-2* fail to form islet α and β cell lineages, and β cell deletion of *Nkx2-2* results in the loss of β cell identity. Consistent with the mouse phenotypes, individuals carrying homozygous mutations in *NKX2-2* develop neonatal diabetes (Flanagan et al., 2014). The lncRNA *βlinc1* (β cell lincRNA1) and its human ortholog *HI-LNC15* are each located in gene deserts between *Nkx2-2* and *Pax1* on chromosome 2 (mice) and chromosome 20 (human), respectively (Arnes et al., 2016). However, unlike *Nkx2-2*, which is expressed throughout the pancreas and in several different endocrine lineages, β*linc1* expression is highly β cell-specific. Furthermore, deletion of the *βlinc1* transcript in mice and siRNA-mediated knockdown of *βlinc1* in MIN6 cell lines, resulted in the down-regulation of *Nkx2-2* and a large subset of *Nkx2-2* β cell targets. ShRNA-mediated knockdown of HI-LNC15 transcript in the human insulin-producing EndoC-βH1 cell line also caused altered expression of genes dysregulated in *βlinc1*^-/-^ mice, suggesting conservation of βlinc1 transcript function in humans. Interestingly, a disproportionate number of *βlinc1-*regulated genes are located on chromosome 2, suggesting that similar to PLUTO, *βlinc1* may function to regulate gene transcription in *cis*. Although the extremely low abundance of *βlinc1* transcript has impaired further biochemical analysis of its molecular activity, *βlinc1* transcript was present in the chromatin fraction and regulated over 70 non-contiguous beta cell-associated genes that are dispersed over a 55 Mb region of chromosome 2, suggesting that the *βlinc1* transcript regulates the expression of a set of functionally related β cell genes through a mechanism that remains to be elucidated.

## Conclusion

LncRNAs are emerging as important regulators of cell-specific gene expression. In pancreatic islets (and in other cell types), genes encoding for critical developmental proteins such as tissue-specific TFs are often associated with antisense divergent or adjacent intergenic lncRNA (Morán et al., 2012; Luo et al., 2016). This suggests that lncRNAs may regulate many key TFs. *Cis*-regulation of tissue-specific TFs by lncRNAs is likely to be a widespread mechanism that enables fine-tuning of TF expression. Furthermore, since lncRNAs tend to be much more cell type-restricted than the associated TFs, lncRNAs may confer cell-specific activities to more broadly expressed TFs. For most cell-specific lncRNAs, however, their possible function remains unknown. Functional interrogation of lncRNAs remains extremely challenging, partly due to their low abundance in cells (Wang et al., 2011; Cabili et al., 2015), and because of our limited understanding on how they should be experimentally approached. Nevertheless, future investigations that provide a systematic functional characterization of β cell-specific lncRNAs promise to identify these previously unsuspected regulators of β cell function, and their potential role in monogenic and polygenic forms of diabetes.

## Author Contributions

BF-C and AB wrote the first draft of the manuscript. LA, JF, and LS wrote sections of the manuscript.

## Conflict of Interest Statement

The authors declare that the research was conducted in the absence of any commercial or financial relationships that could be construed as a potential conflict of interest.
